# Slip, Slop, Slap, Slide, Seek and Sport: A Systematic Scoping Review of Sun Protection in Sport in Australasia

**DOI:** 10.3390/curroncol30010033

**Published:** 2022-12-28

**Authors:** Sarah K. Morton, Simone L. Harrison

**Affiliations:** 1 Skin Cancer Research Unit, College of Public Health, Medical and Veterinary Sciences, Division of Public Health and Tropical Medicine, James Cook University, Douglas, Townsville, QLD 4811, Australia; 2 Royal Brisbane Women’s Hospital, Metro North Hospital and Health Service, Queensland Health, Brisbane, QLD 4029, Australia

**Keywords:** clothing, health promotion, skin cancer prevention, sport, ultraviolet radiation

## Abstract

Australia and New Zealand have the highest incidence of skin cancer. Sport is a fundamental part of Australasian culture, beginning in childhood, often with life-long participation. Participating in outdoor sports can contribute significantly to the lifetime ultraviolet radiation (UVR) dose individuals receive and their risk of developing skin cancer. This systematic scoping review explores the use of sun-protection by outdoor sporting participants in Australasia and considers how sun-protection practices may be improved and better evaluated in the community. A search of electronic databases using the search strategy “sun protection” AND “sport” AND “Australia” yielded 17 studies published in English from January 1992 to August 2021. Study methods included using UV-dosimeters to measure individual UVR-exposure; remote estimates of clothing-adjusted UVR-exposure; direct observation of sun-protection practices; and self-reported sun-exposure and sun-protection. Despite 40 years of ‘Slip, Slop, Slap’ campaigns in Australia, the use of sun-protection in most outdoor sports is inadequate. The paucity of comparable data limited our analyses, demonstrating a need for standardized, objective evaluation tools. Such tools, if used across a range of sports, should inform the development of workable recommendations that sporting clubs could implement and adopt into policy, thus empowering them to better protect the health of their participants.

## 1. Introduction

Skin cancer accounts for the largest number of cancers diagnosed in the Australasian region each year, resulting in significant morbidity and mortality [1]. Age-standardized incidence rates for cutaneous melanoma (CM) in Australia and New Zealand were 36.6 and 31.6 per 100,000, respectively, in 2020, which is more than double to triple the incidence reported for Canada, the United States, and the United Kingdom [1]. In Australia, the age-standardized mortality rate for CM in 2019 was 4.6 per 100,000 [2]. CM is the most common cancer in young Australians, accounting for 15% of all cancers diagnosed in 15–24-year-olds in 2014 [3]. Keratinocyte carcinomas (KC: primarily basal cell carcinomas, squamous cell carcinomas) are the most common cancer diagnosed in Australia, accounting for 959,243 paid Medicare services in 2014 [4]. At least two in three Australians will be diagnosed with skin cancer before the age of 70 [4].

Exposure to ultraviolet radiation (UVR) is the main preventable cause of skin cancer [5]. Populations living in areas with intense ambient UVR and those who work and spend leisure time outdoors in the sun are at increased risk of developing skin cancer [6]. The World Health Organization’s INTERSUN program sought to provide consistency globally by introducing a standard international measurement of UVR, known as the UV-Index (UVI), to frame sun-protection messages [7]. Sun-protection is recommended when the UVI reaches three (moderate) or above (except for prolonged time outdoors) when it is less likely to interfere with maintaining adequate vitamin D levels (serum 25-Hydroxyvitamin D > 50 nmol/L) and potentially compromise bone strength [8].

Outdoor recreational activities and organized sport are fundamental Australian and New Zealand pastimes, and arguably form part of our national identity. Football, Australian Football League (AFL), Netball, Cricket and Touch Football are leading team sports played by 15–24-year-olds in Australia [9]. The popularity of team sports in New Zealand is similar, with Rugby Union, Rugby League, Netball, Cricket, and Football (Soccer) generally considered the leading team sports by participation [10]. These sports are all played outdoors, usually during daylight hours. Many people involved in organized sport spend long periods of time outdoors, often without adequate photoprotection putting them at high risk of sunburn, solar damage, and future development of skin cancer [11]. 

Four decades after the “Slip, Slop, Slap” campaign was launched in Australia, the sun-protective behaviors of sportspeople are still inadequate [12]. The adoption of formal sun-protection programs and policies has been variably successful at an organizational level [13,14,15,16]. Recognition of high-risk activities and behaviors can help identify those who would most benefit from improved sun-protective behaviors [11]. Sporting organizations have been identified as a vehicle for health promotion activities, including sun-protection. 

Current best practice for sun-protective clothing is guided by the Australian standard (AS 4399:2020 Sun-protective clothing – Evaluation and classification) [17]. Garments can be certified with Ultraviolet Protection Factor (UPF) ratings which correspond to classifications of minimum (UPF15), good (UPF30) and excellent (UPF50, 50+) protection against UVR. The original 1996 Australian and New Zealand Standard for sun-protective clothing only measured and reported the transmission of UVR through fabric without considering the design and body surface coverage offered by the garment [18] until the standard was revised in 2017 [19]. Major changes to the sun-protective clothing standard included: (i) introducing body surface coverage requirements; (ii) simplifying the UPF classification scheme; and (iii) setting minimum requirements for specific garments such as hats and gloves [17]. 

In 2019, the consensus statement on sunscreen for Australia and New Zealand recommended daily use of broad-spectrum (chemoprotection against both UVA and UVB) high sun-protection factor (SPF) sunscreen for people living in Australasia when the UVI is forecast to reach 3 or greater [20,21]. Sunscreen should be applied to the face, head, neck, and all parts of the body not covered by clothing, at least 20 min before going outdoors, and frequent re-application is recommended [21]. Maximum protection claimed on sunscreen products is limited to SPF50+ (filters 98% UVR) [21] as per the current Australian and New Zealand Standard for sunscreen products (AS/NZS 2604:2021) [22].

Minimal erythema dose (MED) and standard erythema dose (SED) are the most common radiometric parameters used to quantify UVR-exposure. One MED (200 J/m^2^) is the lowest UVR-exposure to produce perceptible redness (erythema) in previously unexposed human skin [23]. One SED is equivalent to an erythemal effective radiant exposure of 100 J/m^2^ [24].

There is limited data in the published literature regarding the sun-protection knowledge and behavior of participants in outdoor sports in Australasia. There is a need for comparative research, as different sports have distinct norms, including regulations regarding uniforms and clothing requirements, timing (seasonal and diurnal differences) and location of competition (geographic and venue type), and differences in the provision of shade and sunscreen. 

The objective of this study is to systematically review the published literature about the use of sun protection and the level of solar UVR received by outdoor sports participants in Australasia. The factors that influence sun-related behaviors will also be reviewed together with the outcomes of interventions that have been trialed in this setting, providing a basis for formulating recommendations aimed at improving sun-protection in sport in Australasia.

## 2. Materials and Methods

**Figure 1:**
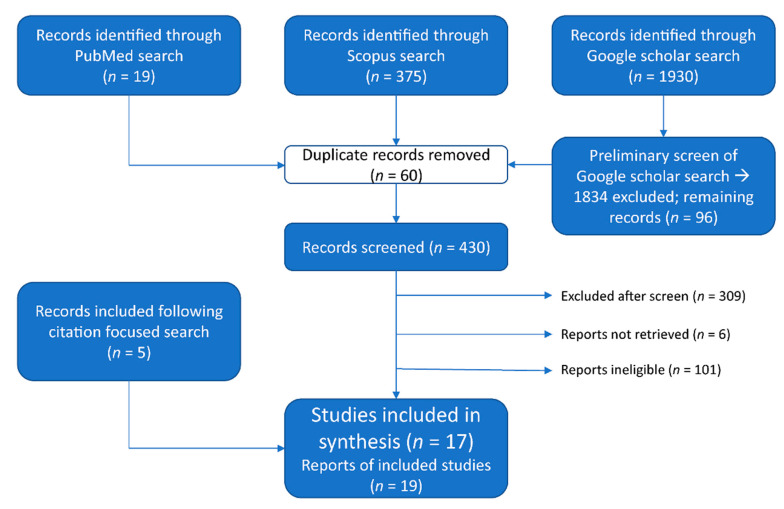
Search strategy as per the 2020 PRISMA guidelines.

**Figure 2:**
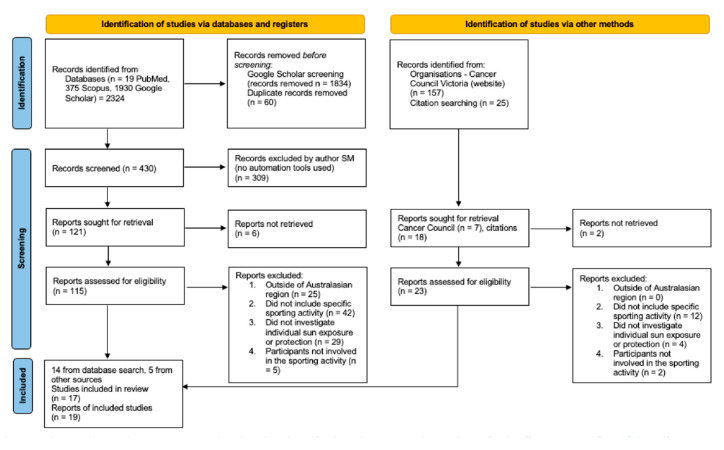
Extended PRISMA Flowchart for Search Strategy [26].

A systematic search of electronic databases was conducted August 2021 in PubMed, Scopus, and Google Scholar using the search strategy: “sun protection” AND “sport” AND “Australia” following the PRISMA protocol for systematic reviews (Figure 1 and Figure 2) [25]. Reference lists of available articles were also reviewed to identify additional relevant citations. The protocol for this systematic review was registered with the Research Registry and its unique identifying number is ReviewRegistry1497 (www.researchregistry.com, accessed on 13 December 2022). 

Included studies were published in English between January 1992 and August 2021, and involved individuals involved in organized recreational or competitive sport in Australasia–comprised of Australia, New Zealand, the island of Papua New Guinea and neighboring islands in the Pacific Ocean. Studies that quantified UVR-exposure, observed or documented self-reported sun-protective behaviors (for the purpose of this review, defined as wearing hats, sun-protective clothing, sunglasses and/or sunscreen) were eligible to be included. Articles were comprised of randomized controlled trials, longitudinal, interventional (case/control), cross-sectional and qualitative studies. Individuals involved in organized sports were defined as participants of the organized sport, including players, coaches, umpires, and sporting officials. 

Articles were excluded if they could not be accessed in English, involved participants outside Australasia, or did not specifically refer to participants in a recognized outdoor sport. Articles exploring sun-protection during ‘physical activity’ in the absence of a named sport were excluded from this review. 

All of the identified articles were reviewed, and abstracted data summarized in tabular format. Given the diversity of articles included, no systematic scoring system was developed to appraise study quality.

## 3. Results

The search criteria yielded 17 studies spanning almost 30 years of investigations into sun-exposure and sun-protection of sports participants within Australia (*n* = 12), New Zealand (*n* = 4) and in both countries (*n* = 1) [23,24,27,28,29,30,31,32,33,34,35,36,37,38,39,40,41,42,43]. Studies were grouped into four general categories: (i) UVR-exposure measured by dosimetry; (ii) UVR-exposure estimated remotely; (iii) observed sun-protective behaviors; (iv) self-reported sun-exposure and sun-protective behaviors (Table 1 and Supplementary Table S1). Sporting activities and events included athletics, bushwalking, cricket, cycling, field hockey, golf, rowing, rugby league, sailing, snow skiing, snowboarding, soccer, surf lifesaving, surfing, swimming, tennis, and triathlon. Most studies examined the sun-protection behaviors of participants in the club/recreational sport environment (*n* = 12), while studies of school sport (*n* = 3) [24,33,34] and elite sport (*n* = 2) [30,43] were less represented. 

Five studies objectively measured the UVR-exposure of sporting participants by dosimetry. Four of these used polysulfone UV-dosimeters [23,27,28,29]. Igoe and co-workers [30] extracted publicly available online data to estimate the UVR-dose received by tennis court staff and players at the Australian Open [30]. Sun-protective behaviors included using sunscreen, clothing, and shade. The four observational studies predominantly recorded clothing-cover [31,32,33,34]. Sunscreen-use was largely self-reported, however one observational study measured sunscreen application by repeatedly weighing freely available sunscreen containers [32]. 

High levels of UVR-exposure were experienced by sport participants [23,24,27,28,29,30]. No more than 1.0 SED daily is recommended for safe UVR-exposure [24,30]. All studies included in this review that calculated SED from dosimetry or estimated UVR-dose remotely, encountered some participants whose exposure exceeded this threshold. High-risk body-sites included the vertex, shoulders, and the back of the hands [23,27,29]. During a 7-day cycling event for charity held during winter in Queensland, average daily exposures exceeded 2.0 SED [23]. High school rowers at a regatta in New Zealand had a median race-time exposure of 1.15 SED, with the highest recorded dose reaching 3.7 SED in a single race [24]. Rowers often compete in multiple races, and thus, would have received significantly higher doses. Recreational golfers in the Darling Downs region were shown to have summertime exposures exceeding 1.0 SED, whereas during the winter months their UVR-exposure fell within safe, daily limits [27]. Comparing the UVR-dose received for a range of outdoor sports, Herlihy and co-workers revealed that sailing crews (17.1 SED), golfers (14.6 SED) and tennis players (8.7 SED) were at highest risk of harmful UVR-exposures, largely owing to the prolonged duration of activity and unshaded locations [29]. Over multiple events and training sessions, triathletes were exposed to extreme UVR-levels, with the maximum UVR-dose reaching 19.1 SED and 21.5 SED during the bicycle stage at Taupo and Busselton triathlons, respectively [28]. Less than one-quarter of students in Dunedin, New Zealand wore sun-protective clothing that covered to below the elbows and knees at their school athletics days when the UV-index was high (>7) [33]. Similarly, only 3.4% of students wore a sun-protective hat [33]. Supervisors’ sun-protection practices were better, with 25.2% wearing a sun-protective hat and 49.3% wearing a shirt with at least elbow-length sleeves [33]. Conversely, 77.3% of student-spectators observed at primary school swimming carnivals in Townsville, Australia, wore sleeved-shirts between events (presumably because of the mandatory swim-shirt policy introduced for Queensland government schools in 2008) [34] while only 30.6% wore a hat [34]. Hat and shirt-use was independent of school size, educational advantage, sun-protection policy score or SunSmart status [34].

New South Wales (NSW) cricket players had high (~90%) sun-protective clothing-coverage, however use of a broad-brimmed or legionnaire hat was uncommon and only 44% had access to shade [31]. Clothing-coverage and access to shade for their coaches was lower at approximately 80% and 20%, respectively [31] (Supplementary Table S1). Approximately half of the cricket clubs endorsed wearing sun-protective clothing, yet most clubs sold or provided baseball caps. Just under half of the clubs had hat-wearing regulations. A non-significant trend towards not wearing a hat was evident in teams without hat-wearing regulations [31]. In another study, 38.4% of retired cricket players from a single club in NSW had been diagnosed with at least one skin cancer, with the highest incidence evident in 45–55-year-olds [40]. Of those with a history of skin cancer, 36.5% reported inadequate use of at least two of the three recommended sun-protection strategies (wear wide-brimmed hat, long-sleeved shirt, and sunscreen) [40].

Combining the use of sunscreen, sunglasses, hat, and shirt coverage, only 14% of triathletes reached the recommended standard of sun-protection expected at SunSmart sponsored triathlons [41]. Snow skiers and snowboarders in Queenstown, New Zealand had high rates (48%) of past sunburn [42]. Although eye-protection was used almost universally among the snow skiers and snowboarders surveyed and 66% of them reported wearing sunscreen, women were significantly more likely than men to wear a protective hat, and wear and re-apply sunscreen [42]. Horsham and co-workers found a more than three-fold increase in sunscreen-use when they intervened at a rugby league carnival in regional Queensland by providing free sunscreen and UVR-detection stickers to 14–18-year-old male and female players, team staff and spectators [32]. 

A large cross-sectional survey examined the sun-protective behaviors of young adults aged 18–30 years, competing in soccer, field hockey, tennis, and surf-lifesaving competitions in South-East Queensland [35,36,37]. Only 20.2% of participants reported adequate sunscreen-use, which was more common in women (80.3%) than men (53.7%) overall, and female soccer and tennis players compared to males [35,37]. Surf-lifesavers reported the highest use of sunscreen at 60.3%, compared with tennis (8.9%), hockey (5.1%) and soccer (4.7%) players overall [36]. Sun-protective clothing, including wearing a hat and sunglasses varied significantly across sports, with more hockey and soccer players indicating uniform and safety regulations prevented them from wearing these [35]. A significantly higher proportion of female tennis and hockey players wore sleeveless tops, than men. Female hockey players were also more likely than males to wear a hat [35]. Most participants competed in environments without shade [35]. History of sunburn during previous sporting seasons was high (69%), with surf-lifesaving participants more likely to have been sunburnt during the last season (88%). Duration of exposure varied across sporting disciplines: hockey players were exposed for the least amount of time on average (88 mins), compared with surf-lifesavers (479 mins) [35]. 

As early as 1999, NSW and Victorian lifesavers reported good sun-protective behaviors [38]. Reported hat-use ranged from 55–89% while wearing a long-sleeved shirt ranged from 60–81%. Reported sunscreen-use was higher at 85–97%, while shade was only used by 62–77% of participants [38]. On cloudy days, all three personal sun-protective measures were less common. Victorian lifesavers had significantly higher levels of all three sun-protective behaviors compared to NSW lifesavers. This difference occurred in the context of long-term sun-protection sponsorship programs being implemented in Victorian surf-lifesaving clubs. All three of these sun-protective behaviors improved among Victorian lifesavers compared to pre-sponsorship findings from 8 years earlier [38].

Surfers were more likely to apply sunscreen in summer (64% to face and 54% to whole body), than in winter, when sunscreen-use halved [39]. 19.1% of surfers reported never applying sunscreen. Wearing rash vests and surf caps was inversely related to temperature, and 224 skin cancers were treated in 14.6% of participants in the year prior to completing the survey [39].

Elite athletes in New Zealand playing rugby, field hockey or rowing reported low levels of sun-protection [43]. Only one of 110 participants reported “always wearing a hat”, while 9% reported always applying sunscreen before sun-exposure [43]. Level of concern about sun-exposure and skin cancer risk differed significantly between elite sporting groups (hockey 82% > rowing 70% > rugby 50%), however it was concluded that their concern was not reflected in their overall sun-protection practices [43]. 

Elite tennis players competing in the Australian Open were exposed to ambient UVR of up to 9.9 SED/hour, with the UVI typically considered “extreme” [30]. The Normalized Clothing Factor (NCF: the relative proportion of the body protected by clothing) was low for players (0.2 no hat; 0.4 with a hat) compared to court staff with NCF-values of 0.6–0.8 [30]. Sun-protection from clothing reduced ambient UVR-exposure to 0.5–1.0 SED/hour for court staff compared to ≤ 2.0 SED/hour for players, demonstrating the effectiveness of the sun-protection policy tournament organizers implemented for court staff [30].

## 4. Discussion

Compliance with recommended sun-protection practices was variable. Despite the number of articles reviewed, there was significant diversity in methods and variables recorded, making comparisons between the available literature challenging. 

Historical standards of sunscreen SPF ratings were reflected over time in the published literature, with many older studies reporting SPF15+ as the threshold for appropriate SPF. Inadequate use or absence of sunscreen was common [35,36,37,39,41,42,43]. The clothing generally worn by most participants in these studies would not be considered compliant in the context of the current Australian (AS 4399:2020) [17] or New Zealand standard (AS/NZS 4399:2017) [44] for sun-protective clothing.

Many athletes considered a suntan to be aesthetically desirable and their sun-protection compliance was influenced by social and group norms [38,41,45]. A socio-ecological approach to promoting sun-safety may help to address these modifiable social cognitions. 

A child’s formative years are the most important in terms of guiding future sun-protective practices. Although childhood sunburn increases melanoma-risk [46,47], relatively little research has specifically assessed the sun-protection practices of primary schoolchildren on the sporting field. Studies examining whether policy and practices that are mandated in the school playground have translated to the sporting field would be valuable.

Optimal performance may be hindered by or perceived to be hindered by increased clothing-coverage. Consequently, uniform and safety requirements dictated by some sporting codes may prevent participants from achieving adequate protection from clothing during competition [35]. A mandatory swim-shirt policy introduced in Queensland Government primary schools in 2008 appeared to be effective in improving the proportion of students observed wearing shirts at inter-school swimming carnivals [34]. Uniformed cricket players and coaches were found to have high levels of clothing-cover, with approximately half of the clubs surveyed consistently endorsing use of sun-protective clothing [31]. Achieving a balance between recommended sun-protective clothing standards [17,44] and clothing that supports optimal sporting performance and participant acceptance will be necessary to maximize uptake. Participants in outdoor sports should be encouraged to apply high SPF, water-resistant sunscreen to any skin not covered by clothing [12], complemented by strategies to increase the accessibility of sunscreen, such as conspicuous placement of free sunscreen dispensers at outdoor sporting venues. The recent wide-spread adoption of free sunscreen dispensers in community settings throughout the USA demonstrates the scalability and sustainability of this initiative [48,49]. A similar approach could be used to augment skin cancer prevention efforts in Australia and New Zealand [12]. If sponsorship is unavailable to cover the cost of sunscreen provision, it could be factored into playing costs and/or spectator entry fees. 

Improved sun-protection practices among Victorian lifesavers reflects the success of sun-protection sponsorship programs as health promotion tools [38]. Status as a role-model may also positively influence sun-protection behaviors [30,38].

Marked differences between sporting disciplines suggest that sport-specific, environmental support may be needed to overcome barriers to sun-protection. Additional factors that may influence this include participants’ age, gender, skin-type, and personal or family history of skin cancer. Those most concerned about skin cancer were more likely to report adequate sunscreen-use [36]. Additionally, many studies report females as more cognizant of the importance of sun-protection, which often translated into females exhibiting better sun-protective behaviors than males [35,36,37,42]. Men’s lower awareness of CM risk [12], poorer skin protection habits [35,36,37,42,50] and higher risk of developing and dying from skin cancer than women [12,51] makes them a priority target-group for skin cancer prevention. One such initiative targeting men’s resistance to sun-protection is the Cancer Council NSW’s “Improve your long game” [12]. This program targets male golfers aged 40+ years by providing NSW golf clubs with educational resources and free sunscreen at the first and tenth tee [12]. Messaging promoting sun-protection to Australian men has also increased recently, as exemplified by the Cancer Council NSW’s 2021 campaign “same goes for you” [52] and the Cancer Council Australia and Australasian College of Dermatology’s 2022 National Skin Cancer Action Week^TM^ campaign [51]. The signage on free sunscreen dispensers also provides an opportunity for targeted messaging to low-use groups such as men [48].

Of the current top five participation sports in Australasia, only cricket and football (soccer) were represented in this literature review. Little is known about the prevalence of sun-protection in AFL, netball and touch football in Australasia.

Wearable UV-dosimeters are a lightweight, cost-effective tool for objectively measuring an individual’s UVR-dose, although post-measurement adjustments should be made to account for clothing-cover in the manner of Igoe and co-workers [30]. UV-dosimetry has been used in a variety of sport settings. Although polymer film dosimetry was the most common type of dosimetry used in the literature we reviewed, biological spore and electronic dosimeters have also been used successfully to measure the UVR-exposure of sport participants [28]. UV-dosimeters are generally positioned on a body-site relevant to the sport involved and typical athlete positioning. Individual SED measurements from UV-dosimetry lack external validity due to posture, dosimeter orientation and varying environmental conditions [11]. Interestingly, all of the UV-dosimetry studies we encountered investigated individual sports, presumably because many team sports are contact sports or have potential for contact during which UV-dosimeters may be damaged, dislodged, re-oriented or their placement altered, interfering with measurements.

Providing UVR-detection stickers is a simple intervention that can improve sunscreen-use and re-application [32,53]. Photochromic molecules form the basis of a UVR-sensitive dye incorporated into a sticker which changes color [53]. When the sticker changes color it serves to remind the wearer to re-apply sunscreen and/or adopt other sun-protective measures. Several UVR-detection stickers are available including “Sundicator” (Treadley Pty Ltd., Gold Coast, Australia) and “SPOTMYUV” (Suncayr, Toronto, Canada) [53]. Low-cost methods for producing UVR-stickers have been described, which should facilitate further research [54].

Remote modeling of UVR-exposure from atmospheric parameters and expected clothing-cover can be used to estimate athletes’ risk, and as a research strategy, minimizes participant-burden and the coordination challenges of field research. Using this method to analyze potential skin cancer risk for individual athletes at the 2020 Tokyo Summer Olympic Games, Downs et al. [55] awarded gold to women’s tennis for highest UVR-exposure. NCF can be estimated by analyzing video footage (NCF = 1 full-body clothing-coverage; NCF = 0 no effective clothing-coverage) [55]. The NCF could be used to compare typical sporting attire worn for a diverse range of outdoor sports in Australasia in a standardized way, using publicly available data/footage. It can be assessed remotely and is more objective than self-reported data. It may enhance the generalizability of results, while avoiding the logistical challenges of UV-dosimetry, including the difficulties associated with quantifying UVR for contact and team sports participants.

Few Australasian studies used standardized scoring-systems for sun-protection [56,57,58]. Dunn et al. [56] developed a ‘compound index of protection’ (CIOP) score to describe the sun-protection of spectators at a cricket test-match. Observation and interview of participants was performed to assess head cover, eye-protection, upper-body cover, and sunscreen-use [56,57]. Similarly, Maddock et al. [58] developed the System for Observing Sun-Protection Factors (SOSPF) to assess beachgoers use of upper-body clothing, headwear, sunglasses, and shade [58]. The benefits of using a scoring-system with well-defined categories include reduced bias and enhanced ability to compare studies. CIOP requires interview of participants to assess sunscreen-use [56], whereas SOSPF can be determined entirely from observation [58]. However, both scores fail to account for lower-body clothing-cover. Numerically scoring multiple components may be challenging in observational studies requiring rapid assessment of participants. Unlike SOSP, sunscreen-use is factored in to CIOP. However, sunscreen re-application is not, but could easily be added.

Sporting organizations have been identified as key stakeholders in health promotion with the ability to deliver sustained public health initiatives from grassroots to elite level. A prominent theme in the available literature was that of sun-protection policy availability, visibility, and implementation. Although articles about the adoption of sun-protection policies in Australasian sporting organizations do not fulfil the inclusion criterion of ‘involving participants of organized sport’, relevant articles of this sort have been summarized in Table 2. These articles demonstrate that the adoption of sun-protection policy varies considerably between sporting organizations in Australasia (Table 2). A recent report [12] suggested that incorporating sun-safety into the policies for “Safe and Inclusive Sport” on the Australian Institute of Sport website [59] and the “Play by the Rules” training courses and support programs available to everyone involved in organized sport including coaches, administrators, officials, players, parents, and spectators [60] may be a cost-effective way to improve sun-safety in outdoor sports. These platforms could also be used to direct sporting clubs to the Cancer Council of Australia’s SunSmart program website which provides a policy template that sporting organizations can adapt [61]. A thorough audit of sun-protection policies in state and regional sporting organizations, and individual clubs would be valuable. Specific areas worth investigating include shade-provision, timing competitions to avoid peak-UVR, and modifying sports uniforms to comply with the current standard for sun-protective clothing [17,44]. Ensuring elite athletes model sun-protective behaviors when competing at international, widely broadcasted competitions would also be invaluable. The 2018 Gold Coast Commonwealth Games was exemplary in sun-protection policy and procedure. They consulted Cancer Council Queensland [62], and researchers from two Queensland Universities (Dr Simone Harrison, James Cook University and Mr. Dean Brough, School of Design, Queensland University of Technology [63]) to ensure uniforms for the 18,000 volunteers and officials were UPF50+ rated and complied with the body-coverage requirements of AS/NZS 4399:2017 [44].

Development and validation of a standardized sun-protection data collection tool and scoring system would facilitate meaningful comparisons between studies. These tools should be developed in accordance with the most recent standards for sun-protective clothing, sunscreen and sunglasses. Methods for objectively recording observed clothing-cover and shade-use are well established, however sunscreen-use is commonly self-reported. Skin swabbing is a noninvasive technique that can detect sunscreen on human skin within a 6-h period [69]. Skin swabbing to objectively determine sunscreen-use would be a valuable addition to recording clothing-cover and shade-use in a standardized field-study data collection tool. Ideally, self-reported data could be collected and validated using skin swabbing [69] to examine the relation between self-reported and verified sunscreen-use while participating in sport. 

Instead of undertaking research in a single sporting activity or event, it is proposed that future studies could involve multiple high-participation sports (e.g., Football, AFL, Netball, Cricket, and Touch Football) and include officials and spectators, in addition to players. Recruitment of local clubs or regional organizations should encompass multiple sites to achieve larger, more representative samples. Sponsorship programs have proven effective in promoting sun-safety by incentivizing participation and supporting local organizations. 

This literature review presents a comprehensive record of research into sun-protection in organized sport in Australasia over 30 years. The diversity of included studies enhanced the overall knowledge gained, but limited comparability between studies and the generalizability of conclusions. Overall, there is a paucity of comparable literature. Studies with self-reported outcomes are intrinsically subjective. Small study populations are vulnerable to selection bias. Recall bias has also been demonstrated in the literature, with individuals’ self-reporting their sun-protection practices more favorably than when they are observed [6]. A systematic quality appraisal was not performed due to heterogeneity of data collection tools and study populations. Despite undertaking a systematic search, it is possible that some relevant literature was missed. It is acknowledged that valuable relevant research has been undertaken outside Australasia, which by virtue of the search criteria, will have been excluded. The decision to limit the review geographically was made primarily to identify current gaps and target areas for future research within Australasia, especially given Australia and New Zealand’s strong sporting culture and high rates of CM and KC [1]. 

## 5. Conclusions

Exposure to UVR is a modifiable risk-factor for skin cancer. Outdoor sporting environments are high-risk UVR- exposure sites. Individuals regularly participating in organized outdoor sport are at-risk for solar damage and skin malignancy secondary to their involvement. Adequate sun-protective behaviors are still lacking despite 40 years of ‘Slip Slop Slap’ health promotion in Australasia. There is a paucity of comparable sun-protection data in sport settings. Future research should incorporate reproducible methods for investigating all elements of sun-protection across a diverse range of sports and sporting environments to produce actionable recommendations for sporting organizations and individual participants. Ongoing policy development and implementation would be valuable from grassroots to all government levels, with the involvement of key stakeholders. 

## Figures and Tables

**Table 1:** Results of search strategy.

Reference(s)	Geographical Location	Sport(s)
UVR-exposure measured by dosimetry:		
Buxton et al. 2021 [24]	Lake Ruataniwha, New Zealand	Rowing
Downs et al. 2009 [27]	Darling Downs region, Australia	Golf
Downs et al. 2020 [28]	Australia and New Zealand	Triathlon
Herlihy et al. 1994 [29]	Hobart, Australia	Swimming, golf, sailing, bushwalking, tennis
Kimlin et al. 2006 [23]	Rockhampton to Brisbane, Australia	Bicycling
UVR-exposure estimated remotely:		
Igoe et al. 2019 [30]	Melbourne, Australia	Tennis
Observed sun-protective behaviors:		
Dobbinson et al. 2005 [31]	NSW cricket clubs, Australia	Cricket
Horsham et al. 2020 [32]	Charleville, Australia	Rugby
McNoe et al. 2016 [33]	Dunedin, New Zealand	Athletics
Turner et al. 2016 [34]	Townsville, Australia	Swimming
Self-reported sun-exposure and sun-protective behaviors:		
Lawler et al. 2007 [35] 2012 [37] Berndt et al. 2011 [36]	Brisbane, Australia	Soccer, field hockey, tennis, surf lifesaving
Dobbinson et al. 1999 [38]	NSW & Victoria, Australia	Surf lifesaving
Meir et al. 2015 [39]	Australia	Surfing
Noble-Jerks et al. 2006 [40]	NSW, Australia	Cricket
Pearson et al. 2004 [41]	Victoria, Australia	Triathlon
Price et al. 2006 [42]	Queenstown, New Zealand	Snow Skiing and snowboarding
Walker et al. 2014 [43]	New Zealand	Rugby, field hockey, rowing

Abbreviations: NSW: New South Wales; UVR: Ultraviolet Radiation.

**Table 2:** Summary of findings regarding sun-protection policy in sports in Australasia obtained from literature published in English between January 1992 and August 2021.

Reference	Locations & Sports Involved	Methods	Findings
Casey et al. 2012 [16]	Victoria, Australia State sporting organizations (SSOs) participating in the Partnership for Health (PfH) scheme (*n* = 25)	Convenience sampling SSO representatives completed Health Promotion and Sport Assessment Tool Audit online	Knowledge of sun-protection policies increased from 62.7% to 80.0%, and sun-protection practices increased from 86.3% to 100.0% following PfH. Compliance to policies increased from 50.0% to 81.3% and practices 64.9% to 84.7%.
Corti et al. 1995 [64]	Western Australia, Sporting organizations sponsored by Healthway May 1991–June 1992, (*n* = 75)	Implementation of Healthway sponsorship, analysis of sun- protection measures	Sun-protection measures in terms of policy increased from 38.7% to 57.3% in sporting organizations; an absolute percentage increase of 18.6% (*p* < 0.001).
Dobbinson et al. 2002 [65] & 2006 [15]	Victoria, Australia Victorian sporting associations	Interview survey with club representative	34% of clubs had sun-protection policies, more prevalent in clubs competing outside in summer months: – diving (86%), lifesaving (81%) and women’s cricket (53%) having highest proportion of clubs with a written sun-protection policy. Water sports were more likely to have written sun-protection policies. Clubs with a written sun-protection policy were significantly more likely to provide portable shade (51%).
Gartland & Dobbinson 2004 [66]	Victoria, Australia Public swimming pools across Victoria (*n* = 208); observation surveys completed (*n* = 205), survey with pool manager (*n* = 185)	Audit of shade structures by trained observers, observation of outdoor staff clothing and zinc use (CIOP calculated) interview of club official	49% of main outdoor pools had no adequate shade. When shade was available over main pools, most provided by ‘natural shade’ (43%). Small number of facilities (3%) had permanent cover over main outdoor pool. 76% of toddler pools shaded in most areas, mostly with permanent shade structures. Interviews with managers suggested that several swimming centers had been active in shade development in recent years, and 41% reported plans to increase shade provided over next 3 years. 28% of centers had written sun-protection policy, 4% in process of developing one. 21% of centers had promotion of sun-protection messages and 16% displayed SunSmart material. 80 centers ran programs for children, where 58% included a component on sun-protection education.
Kelly et al. 2011 [14]	NSW/Canberra, Australia Sporting clubs (*n* = 20) including outdoor soccer, netball, rugby league, outdoor cricket, basketball, athletics/track and field	At each club, one sports official, 10 parents of players (aged 5–14yo) and 5 children (aged 10–14yo) surveyed. Regional sporting association representatives were interviewed over telephone.	Few regional associations had written policies on sun-protection (*n* = 7). Three of these policies were adopted from affiliated state sporting organization. Specified provision/promotion of sunscreen (*n* = 7), appropriate sun-protective clothing (*n* = 7), hats (*n* = 4), disseminating sun-safety information to members (*n* = 6), ensuring adequate shade (*n* = 6), scheduling games outside peak UVR-exposure (*n* = 4), role-modelling good behaviors (*n* = 4). No rugby league, netball or basketball association had a policy on sun-protection. Sponsorship of sports could be a valuable tool to improve sun-protection/promotion of healthy behavior
Kelly et al. 2014 [67]	Australia Australian professionals working in government health and sport agencies *n* = 26	Self-performed questionnaire (online)	Final sample completing all three rounds of survey comprised 8 experts in health promotion, 6 sports management/delivery professionals, 3 experts in physical activity, one expert in nutrition from 4 Australian states and territories. Many of the standards relating to sun-protection were seen to incur additional costs for sports clubs and their members–provision of sunscreen, shade and protective uniforms; some standards unfeasible e.g., use of hats for contact sports & provision of shade at council-owned facilities. Sun-protection was a highly ranked standard for sports clubs to have health promotion activities.
Lawler et al. 2007 [13]	Brisbane, Australia Local sporting club officials from 4 major Australian sports – soccer, tennis, hockey, surf lifesaving	Qualitative audit of policy Face to face interviews with club officials	Formal sun-protection policies less common among hockey, soccer and tennis clubs; some reported informal sun-protection practices. Surf lifesaving clubs had policies developed at state and national level translated into guidelines at club level. Clubs which did have a written policy had implemented comprehensive sun-protection practices. Game duration a factor that influenced perception of skin cancer risk. Common to report limited resources, particularly financial. Lack of shade facilities and control over implementing this is a barrier to sun-protection. Lack of control over timing of competition. Uniform requirements also a barrier to sun-protection. Officials felt that responsibility for sun-protection should be shared by both club and participants.
Potente 2011 [68]	NSW, Australia 3 Local Government Areas in NSW (Sutherland, Gosford, Shoalhaven) Sporting grounds Beach (*n* = 9), pool (*n* = 7), sports grounds (*n* = 8), skate park (*n* = 6)	Audit of shade structures, sun-protection, supportive policies and signage Sites audited by 9 surveyors (in pairs) at two time points	Insufficient shade in 58% of observed sites as sports grounds. Pools were most likely to have shade available over most of the observed areas (36%) and permanent shade structures (75%), however no shade was observed over any outdoor pools. There was only shade over one of the main sporting grounds. Sunscreen was the most popular product available either for free (*n* = 9), or for sale (*n* = 8). All pools had at least one supportive sun-protection policy but only 2 had any related signage.

Abbreviations: CIOP: ‘compound index of protection’ developed by Dunn et al. [56]; *n*: number of sporting venues/organizations/clubs/participants fulfilling the criteria specified in the text of the second column; NSW: New South Wales; PfH: Partnership for Health Scheme; UVR: Ultraviolet Radiation.

## Supplementary Materials

The following supporting information can be downloaded at: https://www.mdpi.com/article/10.3390/curroncol30010033/s1, Table S1: Extended Table of Results [23,24,27,28,29,30,31,32,33,34,35,36,37,38,39,40,41,42,43].
