# Supplementary material for: Slip, Slop, Slap, Slide, Seek and Sport: A Systematic Scoping Review of Sun Protection in Sport in Australasia
Source: Curr Oncol. 2022 Dec 28;30(1):401–15. doi: 10.3390/curroncol30010033 (PMC9858120; doi:10.3390/curroncol30010033)
Supplement: Supplementary file 1 [file curroncol-30-00033-s001.zip › curroncol-2037298-supplementary.pdf]

Supplementary Table S1: Extended Table of Results

Reference	Group characteristics	Methodology	Outcomes
UVR-exposure measured by dosimetry:			
Buxton et al 2021 [24]	Lake Ruataniwha, New Zealand<br>High school rowers (n = 43 rowers)<br>56 race-times included	3-V lithium battery UV electronic dosimeter attached to participant shoulder. Calculation of SED.	Median race-time SED 1.15 SED. Highest SED recorded 3.74.<br>69.6% individual race-time personal exposure exceeded ARPANSA daily recommended SED dose of 1 SED. Median percentage ambient UVR exposure ranged 17-35%.<br>The PE warrants sun-protection. Rowing is a high UVR exposure sport.
Downs et al 2009 [27]	Darling Downs region, Australia<br>Recreational golfers (n = 2 golfers, frequenting 4 golf courses Feb- Aug 2008).	Polysulphone UV-dosimeter attached to participant forearm, back, vertex scalp. Calculation of SED.	Mean summertime exposures 1.4, 2.2 and 3.2 SED at forearm, back and vertex sites, compared to wintertime exposures 0.2, 0.3, 0.5 SED respectively, per round.<br>The PE warrants sun-protection.<br>Increased lifetime risk of developing NMSC playing a weekly round of golf is a result of increased outdoor behavior.
Downs et al 2020 [28]	Australia and New Zealand<br>Triathlon athletes (n = 1 participant each event), (n = 11 total events; 3 training sessions, 8 competitions).<br>Nov 2017-Dec 2018	Polysulphone UV-dosimeter attached to participant swimming cap, bicycle helmet and running cap Calculation of SED.	Ironman events at Taupo (NZ) and Busselton (WA) resulted in highest exposures, during bicycle stage; 19.1 SED and 21.5 SED respectively. Ambient UVR exposure ranged from 3% during a swim leg to 50% during a cycle leg for one triathlon, however for a different triathlon a swim leg recorded 50% ambient UVR exposure. Cycle and running stages often coincide with peak ambient exposure times near solar noon. Triathlon as a sporting discipline can present competitors with extreme UV exposures over an event.
Herlihy et al 1994 [29]	Hobart, Australia<br>N = 94 participants (69 male, 25 female) of swimming, golf, sailing, bushwalking, tennis; aged > 11yo	Polysulphone UV-dosimeter attached to participant cheek, hand, shoulder, back, chest, thigh, calf. Calculation of EED, SED, MED.	Tennis and sailing were subjected to the highest exposure rates, largely due to unshaded location. Tennis exceeding 8.7 SED for up to 2.6h. Golf had a relatively high exposure rate however the golf course used was well shaded.<br>Swimming, where the exposures took place early in the day had the lowest UVR exposure rate.<br>SED scores: tennis 8.7, sailing 17.12, swimming 2.95, walking 8.34, golf 14.6<br>Shoulders had highest UV exposures, followed by back, and hand. Cheeks were least exposed.

Kimlin et al 2006 [23]	Rockhampton to Brisbane, Australia<br>Cyclists in a 7-day charity ride (n = 22), June 2005	Polysulphone UV-dosimeter attached to participant head, dorsal hands, ankles.<br>Calculation of MED<br>Weather conditions extracted	Average daily exposures exceeded 1.0 MED at all body sites except the ankle: ankle 0.94, hand 1.28, head (side) 1.14, head vertex 1.80. Significant difference between MED at (i) ankle and hand, (ii) ankle and head (top), (iii) hand and head (top) and (iv) head (side) and head (top). Mean doses received on the top of the head were significantly higher than all other sites, whereas mean doses received on the ankles were significantly lower than all other sites except the side of the head.<br>Cyclists receive biologically significant doses of UVR, thus sun-protection is warranted.
UVR-exposure estimated remotely:			
Igoe et al 2019 [30]	Melbourne, Australia<br>Australian Open court staff and players	Remotely assesses UV exposures<br>All data extracted from websites (i) match times, duration, uniform and sun-protection rules, (ii) ambient solar erythemal UVR, (iii) weather conditions, (iv) SZA for every minute	Peak UVI values typically above 10 – "extreme" category; ambient UV exposures of up to 9.9 SED per hour. Ambient erythemal UVR exposure for each hour far exceeded ICNIRP occupational exposure limit of 1.0-1.3 SED.<br>Normalised clothing factor (NCF) calculations: chair and line umpire 0.8, ball kid 0.6, player with hat 0.4, player without hat 0.2; court staff required to adhere to sun safe clothing code had better sun-protection and consequent reduction in UVR exposure.<br>Recommendation from Tennis Australia uniform policy to use sunscreen fulfills the necessity of broad range of photoprotective measures. Sun safe role modelling and advertising by court staff.<br>Concluded that tournament organizers had effectively adopted sun-protective practices into uniform policy.
Observation of sun-protective behaviors:			
Dobbinson et al 2005 [31]	NSW cricket clubs, Australia<br>2 weeks mid Feb 2000<br>54 teams observed, 27 matches, (week 1: n = 280 fielding, n = 288 batting), (week 2: n = 310 fielding, n = 302	Observation of clothing worn junior cricket players & coaches<br>Interview with coaches and team managers	56% of players and 80% of coaches had no shade available to them.<br>Average total body cover by clothing was high – 90% for players, 80% for coaches.<br>~2/3 players wore cap for head protection, leaving ears/neck exposed. 26% of players on sidelines and 17% of players fielding did not wear a hat. Use of broad-brimmed or legionnaire hat was uncommon, 7% on sidelines, 23% fielding. Most common leg covering full-length pants for players and majority wore elbow length shirts. 3% of players wore sunglasses.<br>~50% of clubs consistently endorsed use of protective clothing.<br>Non-significant trend towards not wearing a hat in teams without sun policy or hat regulation.

	batting); players <14yo males<br>108 coaches observed<br>32 coaches & 16 managers interviewed.		Concluded that (i) coaches should act as role models for sun-protective behavior and (ii) clubs should develop written sun-protection policies to improve standards, including hat regulations. Broad-brimmed or legionnaire style hats should be made available to both coaches and players.
Horsham et al 2020 [32]	Charleville, Australia<br>Players (14-18yo) and attendees (n = 550) at two-day rugby league carnival	Observation of sunscreen usage with intervention of UV detection sticker<br>Climatic conditions extracted ARPANSA	UVR exposure level consistently high on both days: DAY1 UV 67 SED, DAY2 UV 57 SED; UVI >3 7.0 hrs DAY1, 6.5 hrs DAY2<br>In total 868g of sunscreen used on both days; 19% on Day1 and 81% used on Day 2<br>>3 fold improvement in sunscreen use on DAY2 with provision of UV detection sticker<br>UV detection stickers are a health promotion tool that may improve use of sunscreen in adolescents during sporting events in high UV environments
McNoe et al 2016 [33]	Dunedin, New Zealand Secondary school students and supervisors at school athletics days Feb/Mar 2015<br>N = 1225 students, N = 215 supervisors	Observation of clothing worn, availability of sunscreen and shade, and broadcasting of reminders	All events held when the UV-index exceeded 7 (when sun-protection should be used).<br>Students more likely to wear a sun-protective hat when cloud cover <50% and temperature >20degC.<br>Students: 1.7% wore sunglasses, 3.4% wore a sun-protective hat, 19.0% wore clothing below elbow, 21.4% wore clothing below the knee.<br>Supervisors: 4.2% wore sunglasses, 25.2% wore a sun-protective hat, 49.3% wore clothing below elbow, 59.5% wore clothing below the knee<br>Sunscreen was provided at 5 (50%) of school events, 4 events were sufficiently shaded, and in 2 instances announcement to apply sunscreen was noted.
Turner et al 2016 [34]	Townsville, Australia<br>School students at a swimming carnival 2009 – 2011 & 2015, 41 schools<br>N = 2,916 hat observations<br>N = 2,932 shirt observations	Observation of clothing worn	Median of 30.6% of student spectators wore a hat, 77.3% wore a shirt.<br>Students from non-governmental schools twice as likely to wear a hat (p = 0.003).<br>Hat and/or shirt-wearing behavior not influenced by school size, educational advantage, sun-protection policy score, or SunSmart status.<br>Mandatory swim-shirt policy (introduced in 2008) effective for wearing adequate shirt cover. Hat wearing rates poor.<br>Schoolchildren attending swimming carnivals should not rely on sunscreen or shade.

Self-reported sun-exposure and sun-protective behaviors:			
Lawler et al 2007 [35]<br>Berndt et al 2011 [36]<br>Lawler et al 2012 [37]	Brisbane, Australia<br>18-30yo participating in soccer (n = 65), field hockey (n = 61), tennis (n = 48), surf-lifesaving (n = 63); total n = 237 (140 women, 97 men);<br>Summer 2005/2006	Self-administered cross-sectional questionnaire	29.5% of participants reported no sunscreen use, 47.7% inadequate use, and 20.2% adequate use. Non sunscreen use higher among men compared with women. Surf sports had highest percentage (60.3%) of adequate sunscreen use, compared with tennis (8.9%), hockey (5.1%), and soccer (4.7%).<br>Social cognitive attributes were significantly [p<0.05] associated with inadequate sunscreen use. Adequate sunscreen users had highest levels of concern about skin cancer. Participants with a high sun-protection habit strength had an odds ratio of adequate sunscreen use 17 times that of those with low habit strength.<br>Sunburn during previous sporting season was high at 69%, surf sport participants more likely to have been burnt during the last sporting season (88%). Highest rates of sun-protective behavior in surf sports compared to others.<br>Length of exposure varied: hockey players spent least amount of time (1hr28min), compared to surf lifesaving (7h59min).<br>Surf lifesaving and tennis players had greater hat wearing, this may have been constricted by sporting code rules for soccer and hockey.

Dobbinson et al 1999 [38]	NSW, Victorian Lifesavers<br>NSW (n = 134), Vic (n = 129)<br>Males (67%); >15yo<br>Collected over summer weekends Jan-Feb 1997	Self-administered questionnaire; case-control study	Victorian lifesavers reported more frequent use of hats, long-sleeved shirts and shade shelters whilst on patrol, had more favourable perceptions of themselves as role models for sun-protection and fewer were sunburnt since last patrol.<br>There was improvement in sun-protection in Victorian lifesavers within 10 years of sun-protective sponsorship programs.<br>Attitudes to tanning and sunbaking largely remained the same and were consistent between NSW and Victoria<br>Sun-protection practices have improved in Victorian lifesavers as a result (at least partially) of a sponsorship program, and therefore sponsorships can be effective health promotion tools.
Meir et al 2015 [39]	Australia<br>Surfers 12-67yo residing in Australia, n = 772 participated in survey; full responses n = 685	Self-administered questionnaire (online)	64% “always” or “most of the time” applied sunscreen/zinc to their nose when surfing during warmer months, 54% applied sunscreen to whole body in warmer months, and 19.1% did not apply sunscreen: rates of sunscreen wearing in cooler months approximately half of that in summer months. Wearing rash vests and surf caps was less popular in warmer months.<br>14.6% reported treatment for a skin cancer or lesion in 12 months prior to the survey; a total of 224 separate skin cancers/lesions were reported; most common sites for skin cancers face, hand and forearm; older participants had more skin cancers overall.
Noble-Jerks et al 2006 [40]	NSW, Australia<br>Retired regional representative players of Emu Cricket club, n = 164 players	Self-administered questionnaire	38.4% had been diagnosed with at least one skin cancer with the most common site being the face followed by the arms. 7.9% had skin cancer on 4 or more separate locations; the 45-55yo group had the highest incidence.<br>23 participants of 63 reporting cases of skin cancer reported occasional, very rarely or never used at least 2 of 3 recommended skin protection strategies<br>Concluded that skin protection strategies may help to reduce the risk of skin cancer in cricketers
Pearson et al 2004 [41]	St Kilda, Frankson and Mordialloc, Australia<br>Victorian triathletes 15-61yo<br>N = 206 triathletes (75% male)	Interviewer administered questionnaire at three SunSmart triathlons	54% of participants reported they liked to get a tan; preferred tan was significantly correlated with observed tan.<br>61% used maximum sunscreen SPF 15/15+ usually/always, 10% use sunscreen half the time, 10% use sunscreen sometimes, 19% never/rarely wear sunscreen; athletes who regularly used sunscreen were on average younger compared to those who used sunscreen less often; no gender difference found<br>Singlets most popular top worn (49%) followed by t-shirts (34%). Caps most commonly worn headwear (71%), 28% no hat. 77% of triathletes wore protective sunglasses.

			14% of triathletes reached desired standard of sun-protection – combining use of sunscreen, glasses, a hat and protective shirt. Lack of protective clothing enhances sun exposure risk in the triathlete population.<br>Most participants preferred to warm up/cool down in the shade (49%) or indoors (23%).
Price et al 2006 [42]	Queenstown, New Zealand<br>Skiers and snowboarders at 3 ski fields (Remarkable, Coronet Peak, Cardrona), n = 226, Sep - Oct 2002.	Interviewer administered questionnaire<br>Composite sun-protection index calculated	48% recalled sunburn while skiing or snowboarding in the past. 68% were unaware of any educational messages specific to sun-protection while skiing or snowboarding. Having a skin type resistant to burning and reported awareness of education messages were associated with not using sun-protection<br>Eye protection was employed universally in both groups. 47% of participants adequate sun-protection with sunscreen use 66%, lip balm 78% and reapplication of sunscreen if initially using sunscreen 93%.<br>Women were more likely to practice sun-protective behaviors.
Walker et al 2014 [43]	New Zealand<br>Elite athletes – rugby (n = 35), field hockey (n = 22), rowing (n = 53);<br>Total n = 110 (female n = 34, male n = 76); mean age 23.53±3.11	Interviewer administered questionnaire	Difference between sports those concerned about risk of skin cancer with sun exposure [p = 0.047]; hockey players (82%) > rowers (70%) > rugby players (50%).<br>9% of athletes always apply sunscreen before sun exposure. Significant difference between ethnicity for sunscreen use.<br>Only one athlete reported always wearing a hat, 40 athletes always wore sunglasses and only two athletes reported always seeking shade.<br>Concluded that athletes are concerned about skin cancer, however, are not practicing adequate sun-protection.
